# Atomistic and machine learning studies of solute segregation in metastable grain boundaries

**DOI:** 10.1038/s41598-022-10566-5

**Published:** 2022-04-23

**Authors:** Yasir Mahmood, Maher Alghalayini, Enrique Martinez, Christiaan J. J. Paredis, Fadi Abdeljawad

**Affiliations:** 1 Department of Mechanical Engineering, Clemson University, Clemson, SC 29634 USA; 2 Department of Materials Science and Engineering, Clemson University, Clemson, SC 29634 USA; 3 Department of Automotive Engineering, Clemson University, Greenville, SC 29607 USA

**Keywords:** Atomistic models, Surfaces, interfaces and thin films, Metals and alloys

## Abstract

The interaction of alloying elements with grain boundaries (GBs) influences many phenomena, such as microstructural evolution and transport. While GB solute segregation has been the subject of active research in recent years, most studies focus on ground-state GB structures, i.e., lowest energy GBs. The impact of GB metastability on solute segregation remains poorly understood. Herein, we leverage atomistic simulations to generate metastable structures for a series of [001] and [110] symmetric tilt GBs in a model Al–Mg system and quantify Mg segregation to individual sites within these boundaries. Our results show large variations in the atomic Voronoi volume due to GB metastability, which are found to influence the segregation energy. The atomistic data are then used to train a Gaussian Process machine learning model, which provides a probabilistic description of the GB segregation energy in terms of the local atomic environment. In broad terms, our approach extends existing GB segregation models by accounting for variability due to GB metastability, where the segregation energy is treated as a distribution rather than a single-valued quantity.

## Introduction

Nearly all structural metallic systems are multi-component polycrystalline aggregates; their microstructures are composed of crystalline grains that are internally joined at grain boundaries (GBs). Owing to their local atomic environments, which considerably differ from those in the bulk grains, GBs provide an abundance of sites for the preferential segregation of elemental species in order to lower the total free energy of a metallic alloy system. Numerous experimental^1–6^, theoretical^7–10^, and computational^11–15^ studies demonstrated the impact of GB segregation on materials phenomena and properties, including boundary migration and microstructural evolution^16–18^, transport^19^, wear and friction^20^, embrittlement^21,22^, and activated sintering^4,23^. For example, the treatment by Cahn^24^ quantified the role of GB segregation in mitigating boundary migration. Weissmüller^25^ derived analytical relations for the dependence of GB energy on boundary concentration and alloy thermodynamics. Mishin^7^ examined segregation-induced phase transformations of migrating GBs. The interested reader is referred to the book by Lejček^26^ and references therein for a comprehensive review of studies on GB solute segregation.

While the abovementioned studies provide insights into the impact of GB segregation on many materials processes, the role of GB metastability in solute segregation remains poorly understood. The GB geometry is characterized by eight degrees of freedom (DOF); five are termed macroscopic and the other three are microscopic^27–29^. The macroscopic DOF are divided into three describing the GB misorientation and two defining the GB plane normal^29,30^, whereas the microscopic DOF describe mutual translations of the two abutting grains parallel and perpendicular to the GB plane. In real materials, GBs do not always occupy their ground-state configurations. First, the GB network topology in a polycrystalline material imposes geometric constraints that may drive the boundaries out of their lowest energy states. Second, advances in materials processing have enabled routes to manufacture far-from-equilibrium microstructures, including non-equilibrium GB structures^31^. Several studies examined the impact of GB metastability on boundary properties in pure materials. Vitek et al.^32,33^ and Frolov et al.^34,35^ examined the microscopic DOF and multiplicity of GB structures in pure Cu and body-centered cubic metals, respectively. Han et al.^31^ performed atomistic simulations to examine GB metastability and its statistical properties in Al, Si, and W. Recently, atomic-resolution microscopy demonstrated the coexistence of two different structures of $\Sigma$ 19b GB in pure Cu^36^. However, the impact of GB metastability in solute segregation in metallic alloys has not been systematically examined. Some important questions are relevant here: To what extent does GB metastability affect boundary segregation? And how does it depend on the local atomic environment? If metastability effects are treated as a source of variability in segregation energies, how do we quantify such effects?

The goal of this study is to examine the role of GB metastable structures in boundary segregation. To this end, we leverage atomistic simulations to construct several symmetric tilt GBs. For each GB with prescribed macroscopic DOF, several metastable boundary structures are obtained and used in segregation studies. In this work, the Al–Mg alloy is employed as a model system, as Al–Mg alloys are of great interest in many structural applications^37,38^. Further, segregation of Mg solutes to Al GBs has been experimentally observed^39^. Our atomistic studies are used to correlate segregation energetics with the local atomic environment in metastable GBs. Then, simulation data are used to develop a Gaussian Process machine learning model of GB segregation energy. Gaussian Process modeling is employed, as it allows us to add prior knowledge and update predictions as more data are obtained^40,41^, and it accounts for variability by treating segregation energy as a distribution rather than a single-valued quantity.

## Methods

In this work, atomistic simulations employing Molecular Statics (MS) and Molecular Dynamics (MD) are used to examine the impact of GB metastability on solute segregation in a model Al–Mg alloy. All atomistic simulations reported in this work are performed using the Large-scale Atomic/Molecular Massively Parallel Simulator (LAMMPS) package^42^ and visualizations of atomistic structures are generated using OVITO^43^. The OVITO implementation of the adaptive common neighbor analysis (CNA) algorithm^44^ is used to reveal face-centred cubic (FCC) ordering and identify materials defects, i.e., GB regions. OVITO is also used to perform Voronoi analysis to quantify the local atomic environment. With such a calculation, Voronoi tessellation of the atomistic simulation box is performed using the atomic positions as cell centers, and this process yields the Voronoi atomic volume.

**Figure 1:**
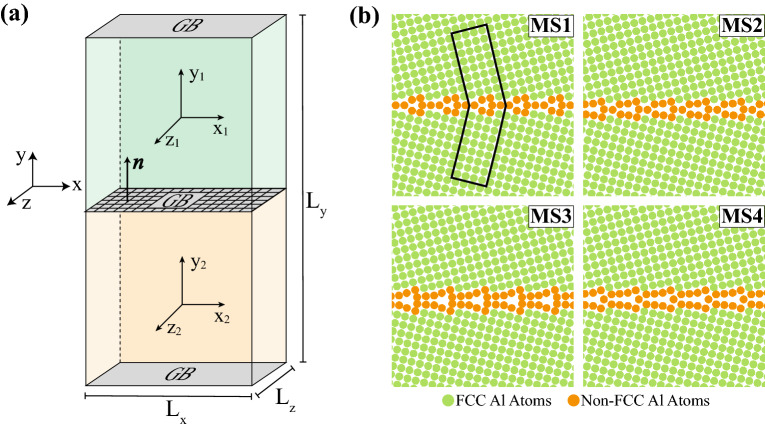
(**a**) The bicrystal geometry employed in this work. (**b**) The four structures of the $\Sigma 17$ [001] STGB, where green (orange) denote FCC (non-FCC) atoms according to CNA. In (**b**), the region enclosed in black line in the MS1 GB structure encompasses atomic sites considered for Mg swaps and segregation studies. The same approach is used for all GB structures explored in this study.

### Generation of metastable grain boundaries

A series of Al bicrystals with [001] and [110] symmetric tilt GBs (STGBs) are generated and their segregation behavior is examined using an embedded-atom method (EAM) interatomic potential that was fit using ab initio and thermodynamic data of properties, such as alloy phase diagram and vacancy interaction energies^45^. A total of eleven [001] and eleven [110] STGBs are considered in this study. Al bicrystal geometries with planar GBs are generated using the $\gamma$-surface method^46^. For each bicrystal, a fully periodic Al system is created from two half crystals, each of which is rotated such that the resulting planar GB between the half crystals has the prescribed misorientation angle. Figure 1a shows a schematic representation of the bicrystal geometry used in this work, where the GB plane normal $\mathbf{{n}}$ aligns with the *y*-axis of both crystals. Table 1 lists the *y*-axis for the upper ($\mathbf {y_1}$) and lower ($\mathbf {y_2}$) crystals for each of the [001] and [110] STGBs examined in this work. The target dimensions of the bicrystal geometry are varied in order to accommodate an integer number of unit cells necessary to capture the periodicity of each GB atomic structure. The thickness along the *z*-direction, $L_z$, of the atomistic systems with [001] GBs is set to 20.23 Å, while that for [110] GBs is 28.60 Å. The reader is referred to Supplementary Table S1 online for the dimensions of the simulation box and number of atoms for each bicrystal system.

**Figure 2:**
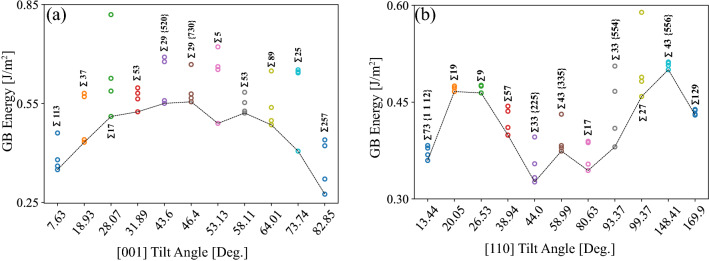
GB energy as a function of the misorientation angle for the (**a**) [001] and (**b**) [110] STGBs. In both panels, the symbols indicate the energy of the metastable GB structures used in this work and the dashed lines trace the energy of the lowest 0 K (i.e., MS1) structures.

For each GB with prescribed macroscopic DOF, a sequence of relative displacements $\mathbf{{t}} \equiv (t_x, t_y, t_z)$ between the upper and lower half-crystals is used in conjunction with atom deletions and conjugate gradient energy minimizations, while allowing the simulation box to expand or contract in the perpendicular direction to the GB plane. As a result, $t_y$ along the GB normal is set by the conjugate gradient energy minimization step and $(t_x, t_z)$ defines the relative in-plane displacements. The process of exploring the space of relative displacements $(t_x, t_z)$ and atom deletions has been widely used to mine for the GB structure with the lowest 0 K boundary energy^47,48^, and is used in this work to generate metastable GBs. Four GB structures with distinct relative displacements and different 0 K energies are selected for each GB misorientation and labeled MS1, MS2, MS3, and MS4, where MS1 denotes the structure with the lowest 0 K energy. Figure 1b shows one example of the four metastable structures for the $\Sigma 17 [001]$ STGB with 0 K energies of 0.51, 0.59, 0.63, and 0.82 J/m$^2$ for the MS1, MS2, MS3, and MS4 structures, respectively. Figure 2a, b show respectively plots of the energies of the four metastable structures for the [001] and [110] STGBs employed in this work. In both panels, the dashed line in the plot traces the lowest energy GB structures as obtained using the $\gamma$-surface method. The trends for the lowest GB energy structures are consistent with prior studies of [001]^49^ and [110]^50^ STGBs.

After the generation of the metastable GB structures, the atomistic bicrystal systems are annealed at 77 K for 50 ps in the NPT ensemble applying a Langevin thermostat^51^ and Berendsen barostat^52^ to achieve zero pressure along the GB plane normal direction. Cryogenic materials processing has been used to manufacture Al-based alloys^53^. Then, the systems are brought back to 0 K over 50 ps followed by another conjugate gradient energy minimization. This annealing step is performed to ensure that all GB structures employed in this work are metastable at least with respect to this low temperature anneal, i.e., avoiding the use of structures that are in an unstable equilibrium state.

**Table 1:** Crystallographic orientations of the two Al half crystals and the resultant misorientation angle for all [001] and [110] GBs explored in this work. Included are the 0 K energies of the metastable GB structures. MS1 corresponds to the lowest energy structure.

Tilt	$\Sigma$	$\mathbf{{y}}_1$ / $\mathbf{{y}}_2$	$\theta$	MS1	MS2	MS3	MS4
Axis		[hkl]$_{\text {U}}$ / [hkl]$_{\text {L}}$	$(^{\circ })$	0 K Energy (J/m$^2$)			
[001]	113	[15 1 0] / [15 $\bar{1}$ 0]	7.63	0.350	0.361	0.380	0.461
	37	[7 5 0] / [5 7 0]	18.93	0.433	0.441	0.571	0.581
	17	[4 1 0] / [4 $\bar{1}$ 0]	28.07	0.511	0.588	0.626	0.819
	53	[7 2 0] / [7 $\bar{2}$ 0]	31.89	0.525	0.564	0.581	0.597
	29	[5 2 0] / [5 $\bar{2}$ 0]	43.60	0.550	0.559	0.677	0.691
	29	[7 3 0] / [7 $\bar{3}$ 0]	46.40	0.556	0.567	0.579	0.668
	5	[3 1 0] / [1 3 0]	53.13	0.490	0.653	0.662	0.722
	53	[9 5 0] / [9 $\bar{5}$ 0]	58.11	0.521	0.527	0.553	0.584
	89	[8 5 0] / [8 $\bar{5}$ 0]	64.01	0.485	0.498	0.515	0.649
	25	[4 3 0] / [4 $\bar{3}$ 0]	73.74	0.406	0.625	0.646	0.652
	257	[17 15 0] / [17 $\bar{15}$ 0]	82.84	0.275	0.321	0.422	0.440
[110]	73	[1 $\bar{1}$ 12] / [$\bar{1}$ 1 12]	13.44	0.399	0.411	0.436	0.444
	33	[1 $\bar{1}$ 8] / [$\bar{1}$ 1 8]	20.05	0.381	0.409	0.467	0.506
	19	[1 $\bar{1}$ 6] / [$\bar{1}$ 1 6]	26.53	0.464	0.475	0.476	0.476
	9	[1 $\bar{1}$ 4] / [$\bar{1}$ 1 4]	38.94	0.500	0.507	0.511	0.512
	57	[2 $\bar{2}$ 7] / [$\bar{2}$ 2 7]	44.00	0.466	0.472	0.475	0.475
	33	[2 $\bar{2}$ 5] / [$\bar{2}$ 2 5]	58.99	0.374	0.379	0.383	0.431
	43	[3 $\bar{3}$ 5] / [$\bar{3}$ 3 5]	80.63	0.344	0.354	0.387	0.389
	17	[3 $\bar{3}$ 4] / [$\bar{3}$ 3 4]	93.37	0.459	0.482	0.489	0.589
	43	[5 $\bar{5}$ 6] / [$\bar{5}$ 5 6]	99.37	0.362	0.333	0.354	0.396
	27	[5 $\bar{5}$ 2] / [$\bar{5}$ 5 2]	148.41	0.430	0.431	0.438	0.439
	129	[8 $\bar{8}$ 1] / [$\bar{8}$ 8 1]	169.90	0.339	0.342	0.365	0.370

### Grain boundary solute segregation

Once the four GB structures for all [001] and [110] STGBs are generated, they are then used in our Mg segregation studies. For each GB system, an Mg atom is placed in the bulk grain far from the GB (i.e., typically $L_y/4$ away from the GB plane). Then, we perform conjugate gradient energy minimization of the system with Mg in the bulk while allowing the system to expand or contract along the GB plane normal direction and record the system’s total energy $E_{bulk}$. Following the approach of Hoagland and Kurtz^54^, we identify the repeat structural unit for each GB (i.e., in general a three-dimensional (3D) polyhedron) and construct a 3D atomistic region encompassing the repeat unit and extending $\approx 20$Å  into the bulk regions on each side of the GB and along the *z*-direction of the atomistic systems. Figure 1b shows an example for the MS1 structure of the $\Sigma 17$ [001] STGB, where the region enclosed by the solid line encompasses the repeat structural unit for this GB. Once these regions are identified for each metastable GB structure and for all boundary misorientations, they are used to perform atomic swaps with the Mg atom in the bulk as follows: (i) Each atom within this region is swapped with the Mg atom in the bulk; (ii) conjugate energy minimization is performed while allowing the simulation box to expand or contract in the perpendicular direction to the GB plane; and (iii) the energy of the relaxed system with the Al at the GB site *i*, $E^{(i)}_{gb}$, is then recorded. The segregation energy $\Delta E^{(i)}$ of this specific site *i* within the selected GB structure is obtained as:

1 $$\begin{aligned} \Delta E^{(i)} = E^{(i)}_{gb} - E_{bulk}. \end{aligned}$$

Again, this process is repeated for each atomic site *i* within the identified 3D region for each of the four metastable GB structures and for all STGBs explored in this work. With the definition given by Eq. (1), $\Delta E^{(i)} < 0$ indicates preferential GB solute segregation, while $\Delta E^{(i)} > 0$ indicates the tendency to desegregate. In addition, using the atomic Voronoi volume for a site *i*, $V^{(i)}$, and the one for an Al bulk lattice site, $V_o$, we define the relative atomic excess volume $\Delta V/ V_o$ as:

2 $$\begin{aligned} \frac{\Delta V}{V_o} = \frac{V^{(i)} - V_o}{V_o}. \end{aligned}$$

The use of $\Delta V/ V_o$ is motivated by the definition of cubic dilatation $\Delta V/ V_o = J - 1$^55^, where *J* is the Jacobian that quantifies the difference in volume between two states.

### Gaussian process regression

In recent years, machine learning (ML) has gained significant interest in materials design and discovery efforts, as it presents computationally tractable tools to construct surrogate models to complement physics-based computational and/or experimental studies^56^. In this work, the atomsitic data for GB segregation are used to construct a ML Gaussian Process model relating GB segregation energy to the atomic excess volume $\Delta V/ V_o$. Herein, we present the salient features of Gaussian Process Regression (GPR) modeling. For further details, the reader is referred to Ref.^57^ GPR is a probabilistic non-parametric Bayesian ML modeling approach. Specifically, in this paper, model outputs, *y*, are assumed to be related to inputs, *x*, through a Gaussian process, *f*(*x*), with a mean function $\mathbf {\mu }(x)$ and a squared exponential covariance function, $k_{SE}(x,x')$:

3a $$\begin{aligned} y&= f(x)+\varepsilon , \end{aligned}$$

3b $$\begin{aligned} f(x)&\sim GP(\mathbf {\mu }(x),k_{SE}(x,x')), \end{aligned}$$

3c $$\begin{aligned} \varepsilon&\sim \mathcal {N}(0,\sigma _{n}^2), \end{aligned}$$

3d $$\begin{aligned} k_{SE}(x,x')&= \sigma _f^2\exp \left( -\frac{|x-x'|^2}{2l^2}\right) , \end{aligned}$$

where $\sigma _f$ and *l* are the GPR hyperparameters that control the variance and length scale of the Gaussian process, respectively. The model also includes a variability term $\varepsilon$, which is modeled as a zero-mean white Gaussian noise process with variance $\sigma _{n}^2$.

Using an uninformed prior, the values for the hyperparameters and noise variance are derived from training data, ${\{X,\mathbf{y} \}}$, in which $X = (x_1, ..., x_n)$ is a vector of the *n* $\Delta V/V_o$ input points and $\mathbf{y} = (y_1, ..., y_n)$ is a vector of the corresponding GB segregation energy $\Delta E^{(i)}$ output. For a given a vector of test cases $X_*$, the corresponding GPR prediction is then characterized by a random variable $\mathbf{f} _*$ with mean and variance^57^:

4a $$\begin{aligned} \overline{\mathbf{f }}_*&= K(X_*, X)[K(X,X) + \sigma _{n}^2I]^{-1} \mathbf{y} , \end{aligned}$$

4b $$\begin{aligned} cov(\mathbf{f} _*)&= K(X_*,X_*) - K(X_*, X)[K(X,X) + \sigma _{n}^2I]^{-1}K(X, X_*). \end{aligned}$$

where $K(\cdot ,\cdot )$ is the covariance matrix corresponding to covariance kernel $k_{SE}$, and *I* is the identity matrix of size *n*. To determine the predictive power of the GPR model, the atomistic data is randomly divided into two groups $\{X,\mathbf{y} \}$ and $\{X_*,\mathbf{y} _*\}$ of size *n* and $n_*$ used for training and testing, respectively. After training the model based on $\{X,\mathbf{y} \}$, the cross-validation root mean square error (RMSE) is determined using the testing set with size $n_*$ as:

5 $$\begin{aligned} \text {RMSE} = \sqrt{\frac{1}{n_*} \sum _{j=1}^{n_*}(y_{j} - \overline{\mathbf{f _*}}(x_{j}))^2 }, \end{aligned}$$

where $\{x_j,y_j\}$ and $\overline{\mathbf{f _*}}(x_{j})$ represent the *j*th test data point and corresponding GPR prediction mean, respectively^58^.

## Results and discussion

**Figure 3:**
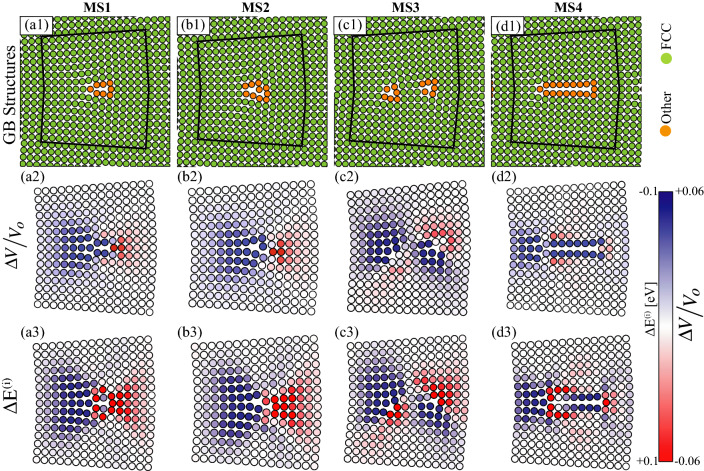
The four structures of the $\Sigma 113$ [001] (15 1 0) GB. (**a1**–**d1**) Atomic structures, where green (orange) indicate FCC (defect) atoms according to CNA. Sites within the regions outlined in black are used in Mg segregation studies. Close-up views of the regions enclosed in black lines, where atoms are colored according to: (**a2**–**d2**) $\Delta V/ V_o$, where blue (red) indicates large (small) atomic volume; and (**a3**–**d3**) Mg segregation energy $\Delta E^{(i)}$, where red (blue) indicates $\Delta E^{(i)} > 0$ ($\Delta E^{(i)} < 0$).

We start our analysis by exploring the atomic structures of the metastable Al GBs and their impact on Mg segregation. Figure 3 shows representative results for the four structures of the $\Sigma 113$ [001] (15 1 0) STGB, where again the structure labeled MS1 corresponds to the lowest energy configuration as obtained using the $\gamma$-surface method. The atomic structures of the four GBs are shown in Fig. 3a1–d1, where green (orange) indicates FCC (non-FCC) environments according to CNA. It can be seen that the structures labeled MS1 and MS4 are symmetric with respect to the GB plane, while the ones labeled MS2 and MS3 are asymmetric. As shown in Table 1, the 0 K energy for the MS1, MS2, MS3, and MS4 structures of this GB are 0.35, 0.36, 0.38, and 0.46 J/m$^2$ (i.e., 30% variation between the smallest (MS1) and largest (MS4) GB energy). In Fig. 3a1–d1, the areas enclosed by the solid black lines are 2D views of the regions used in the Mg segregation studies for these GB structures.

Using the Voronoi structural analysis in Ovito, Fig. 3a2–d2 shows respectively the atomic structures of the MS1, MS2, MS3, and MS4 systems of the $\Sigma 113$ [001] GB colored by $\Delta V/ V_o$, where blue (red) indicates expansion (contraction) corresponding to an atomic volume that is larger (smaller) than that in the bulk lattice. The asymmetry in the structural units of these GBs is also reflected in $\Delta V/ V_o$ contours. For example, Fig. 3c2 shows a spatial distribution of $\Delta V/ V_o$ that is asymmetric with respect to the GB plane. While this GB is macroscopically formed by symmetric orientations of the upper and lower crystals, it is observed that variations in the GB atomic structures driven by the microscopic DOF lead to the observed spatial variations and asymmetry in the atomic volume. To correlate the local variations in $\Delta V/V_o$, due to GB metastability, with the segregation energy, Fig. 3a3–d3 shows the corresponding atomic structures, where each atomic site is colored by the Mg segregation energy $\Delta E^{(i)}$ at that site. Here, blue (red) represents negative (positive) energy, indicating a tendency to segregate (desegregate). A close examination of Fig. 3a2–d2 and a3–d3 reveals that atomic sites with preferential Mg segregation are ones with large free volume (i.e., blue in the figures corresponds to sites with large atomic volume and negative segregation energy), and that this trend exists in all metastable structures of this GB. It is also observed that for this low angle STGB the preferential sites for Mg segregation are not confined to GB core atoms, but extend spatially to regions nearly 1-2 nm into the bulk crystals.

**Figure 4:**
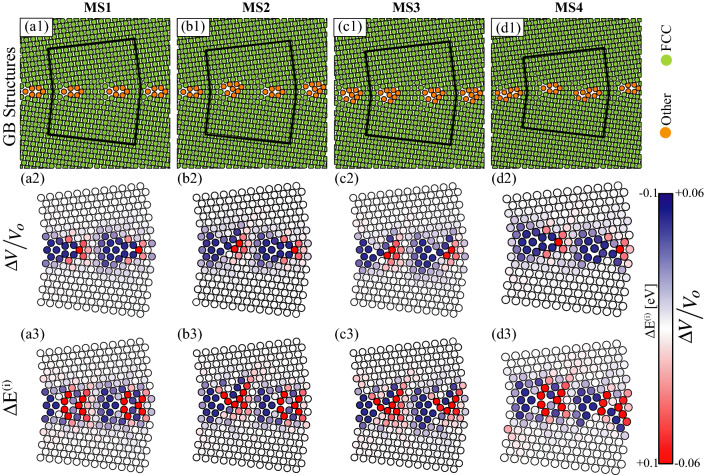
The four structures of the $\Sigma 73$ [110] (1 1 12) GB. (**a1**–**d1**) Atomic structures, where green (orange) indicate FCC (defect) atoms according to CNA. Sites within the regions outlined in black are used in Mg segregation studies. Close-up views of the regions enclosed in black lines, where atoms are colored according to: (**a2**–**d2**) $\Delta V/ V_o$, where blue (red) indicates large (small) atomic Voronoi volume; and (**a3**–**d3**) Mg segregation energy $\Delta E^{(i)}$, where red (blue) indicates $\Delta E^{(i)} > 0$ ($\Delta E^{(i)} < 0$).

The qualitative results in Fig. 3 for the $\Sigma 113$ [001] have also been observed in [110] STGBs. For example, Fig. 4 shows the results for the four structures of the $\Sigma 73$ [110] (1 1 12) STGB, where Fig. 4a1–d1 depicts the atomic configuration of the four structures of this GB, where green (orange) indicates FCC (defect) atoms according to CNA. It can be seen that the structural unit of the MS1 boundary (Fig. 4a1) is symmetric, whereas metastable MS2, MS3, and MS4 systems (Fig. 4b1–d1) have asymmetric structural units. Figure 4a2–d2 shows the atomic structures colored by $\Delta V/V_o$, where blue (red) indicates expansion (contraction) corresponding to an atomic Voronoi volume that is larger (smaller) than the one in the bulk lattice. Similar to the results depicted in Fig. 3, GB metastability influences both the magnitude and spatial distribution of segregation energy, which is shown in Fig. 4a3–d3. Again, a close examination of Fig. 4a2–d2 and a3–d3 shows that negative $\Delta E^{(i)}$ values are correlated with sites with large $\Delta V / V_o$ values. Further, due to the asymmetry in the GB structural units (e.g., metastable MS2, MS3, and MS4), the GB segregation behavior is also asymmetric. For example, a close examination of the MS3 metastable structure (Fig. 4c1 and c3) shows that the preferential sites for Mg segregation are asymmetric with respect to the GB plane.

**Figure 5:**
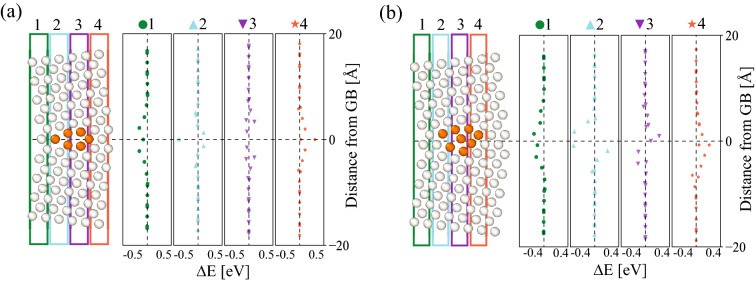
For the $\Sigma 73$ [110] GB, segregation energy line scans across the (**a**) symmetric MS1 (see Fig. 4a1) and (**b**) asymmetric MS2 (see Fig. 4b1) structures. Four line scans are shown, each with a width of 4Å.

To further demonstrate this asymmetry effect in Mg segregation, Fig. 5a, b show respectively several line scans of segregation energies across the symmetric MS1 (see Fig. 4a1) and asymmetric MS2 (see Fig. 4b1) structures of the $\Sigma 73$ [110] GB, where the line scan width is set to 4 Å. Symmetric GB segregation energies with respect to the GB plane are observed for all line scans in the MS1 structure. On the other hand, the MS2 structure exhibits asymmetric segregation profiles, and this observed segregation asymmetry varies along the GB plane, see Fig. 5b. Experimental studies revealed asymmetric segregation and uneven solute distributions across a wide range of GBs in systems such as Fe-Si^59^ and Mg-based^60^ alloys. A very recent study by Alkayyali and Abdeljawad^8^ showed that the asymmetry in GB segregation greatly influences dynamic solute drag behavior of doped GBs.

**Figure 6:**
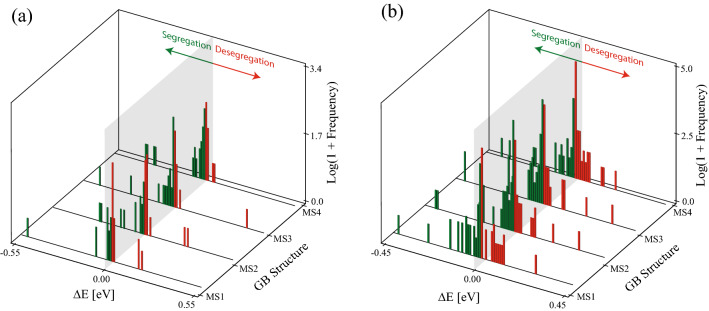
On a semi-log scale, histograms of the Mg segregation energy for the four structures of the (**a**) $\Sigma 5$ [001] (310) and (**b**) $\Sigma 19$ [110] (116) STGBs. The shaded plane in gray marks the zero segregation energy plane. Regions in green (red) indicate segregation (desegregation).

The results depicted in Figs. 3, 4, 5 can be quantified by constructing histograms of the segregation energy for each GB metastable structure and for all GB misorientations. As a demonstration, Fig. 6a, b show respectively histograms, with a bin size of 0.01 eV and on a semi-log scale, of Mg segregation energy for the four structures of the $\Sigma 5$ [001] (310) and $\Sigma 19$ [110] (116) GBs, where regions in green (red) indicate segregation (desegregation). In both panels, the shaded plane in gray marks the zero segregation energy. The reader is referred to Supplementary Fig. S1 for a plot of the data in Fig. 6 using GB site count instead of the natural log of the frequency. It can be seen that the MS1 and MS2 structures of the $\Sigma 5$ [001] (310) GB (see Fig. 6a) favor Mg desegregation, as the number of sites with $\Delta E > 0$ is larger than the ones with $\Delta E < 0$. However, this behavior is different for the MS3 and MS4 structures of this GB, where it can be seen that the fraction of preferential Mg segregation sites is increased compared to the MS1 and MS2 structures. Further, it can be seen that the four structures of the $\Sigma 19$ [110] (116) GB shown in Fig. 6b exhibit larger fraction of sites with preferential Mg segregation compared with the structures of the $\Sigma 5$ [001] (310) GB shown in Fig. 6a. The MS2 and MS3 metastable structures of the $\Sigma 19$ [110] (116) GB are characterized by a larger fraction of sites with preferential Mg segregation compared with the MS1 lowest energy structure. The results depicted in Fig. 6 suggest that GB metastability influences Mg segregation behavior by altering the number of GB atomic sites with preferential segregation.

**Figure 7:**
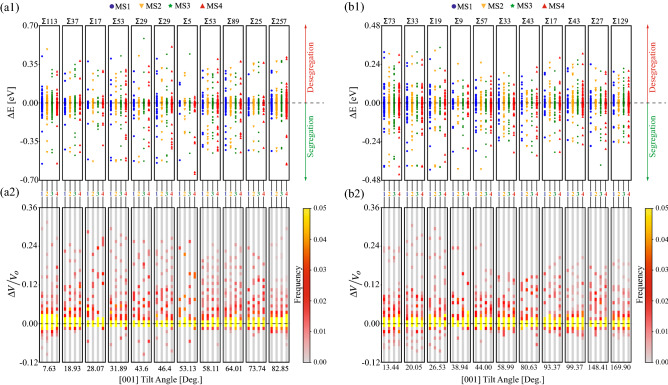
[(**a1**) and (**b1**)] Segregation energy $\Delta E$ and [(**a2**) and (**b2**)] $\Delta V / V_o$ distributions for the (**a**) [001] and (**b**) [110] STGBs as a function of GB misorientation. $\Delta E$ data for each metastable structure are assigned a unique color.

Next, we extend the results in Fig. 6 to all GBs examined in this study. To this end, the segregation energy and $\Delta V / V_o$ data for all four structures of all GBs are used to construct distributions, and the results are shown in Fig. 7 as a function of GB misorientation angle. Figure 7a1, a2 show respectively segregation energy and atomic Voronoi $\Delta V / V_o$ distributions for [001] STGBs. The segregation energy and $\Delta V / V_o$ distributions for [110] STGBs are shown in Fig. 7b1, b2, respectively. Figure 7 reveals that for [001] (see Fig. 7a1) and [110] (see Fig. 7b1) STGBs metastability influences the number of available sites for segregation. For example, the MS4 metastable structure of the $\Sigma 5$ [001] GB has sites with more negative segregation energy (see Fig. 6a1) compared with the other structures of this GB. It is also observed that GB metastability results in structures with atomic sites that vary considerably in their $\Delta V/V_o$ and segregation energy values. As expected, the histograms of $\Delta V/V_o$ in Fig. 7a2, b2 are skewed to positive $\Delta V/V_o$ values, as the GB regions are characterized by excess free volume. While the atomistic data in Fig. 7 show a plethora of possible segregation sites for the various metastable GB structures, no clear dependence on GB misorientation can be deduced. This is expected as our segregation and $\Delta V/V_o$ data describe local per atomic site quantities, while the GB misorientation provides a macroscopic characterization of the GB geometry.

**Figure 8:**
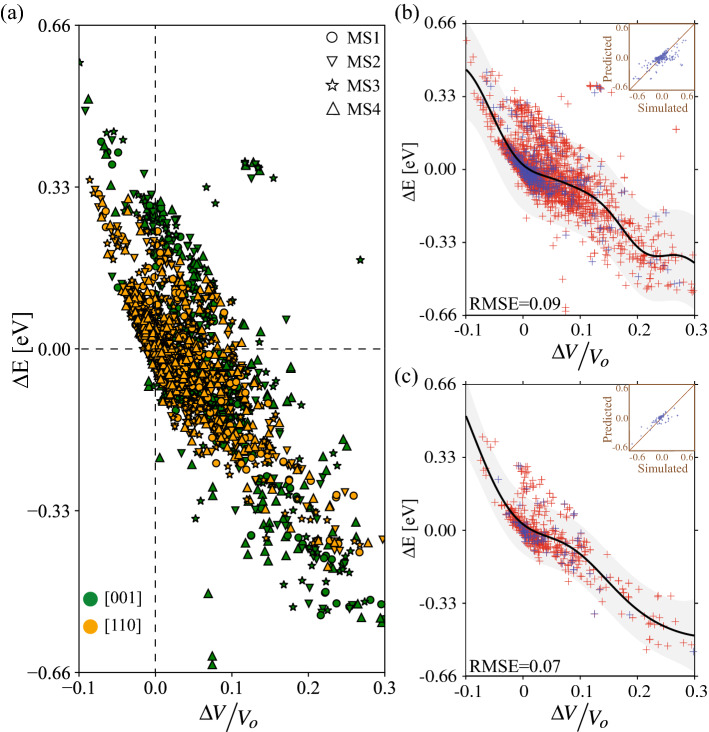
(**a**) GB segregation energy as a function of $\Delta V/V_o$ for all metastable structures and for all GBs explored in this work. The ML GPR model of segregation energy $\Delta E$ as a function of $\Delta V/V_o$ using atomistic data of (**b**) all metastable (i.e., MS1, MS2, MS3, and MS4) and (**c**) only the lowest GB energy (i.e., MS1) structures. In (**b**) and (**c**), the solid line indicates the predicted mean and the shaded region corresponds to the 95% confidence interval. Data points in red (blue) denote training (testing) sets. The insets in (**b**) and (**c**) depict cross-validations, where GPR predictions are plotted against the atomistic data.

Finally, we plot the GB segregation energy as a function of $\Delta V/V_o$ for all metastable structures and for all GB misorientations, and the results are shown in Fig. 8a. Data points in green (orange) denote [001] ([110]) STGBs and different marker shapes are used for the various metastable GB structures. A clear trend can be seen, where the segregation energy decreases (i.e., preferential Mg segregation to Al GBs) with increasing $\Delta V/V_o$—large $\Delta V/V_o$ values indicate sites with large atomic Voronoi volume compared to the one in the bulk crystal. This suggests that for the Al–Mg system, the segregation of Mg to Al GBs is elastically driven (i.e., atomic sites with large Voronoi volume are preferential sites for Mg segregation), at least in the dilute limit. It is also interesting to note that the correlation of GB segregation energy with $\Delta V/V_o$ is evident for all GB metastable structures.

The data in Fig. 8a are used to construct a ML GPR model of GB segregation energy as a function of $\Delta V/V_o$. To this end, the atomistic simulation data are randomly split such that 90% (10%) of data are used to train (test) the GPR model. The training data, where $\Delta V/V_o$ is used as the predictor for the GB segregation energy $\Delta E$, are employed to fit and obtain the hyperparameters of the GPR model. To demonstrate the role of GB metastability, two GPR models are constructed; the first employs data from all GB metastable structures (i.e., all MS1, MS2, MS3, and MS4 structures for all GB misorientations) and the second uses the data of only the lowest energy (i.e., MS1) structures. Figure 8b, c show respectively GPR model predictions using all GB metastable structures (Fig. 8b) and only the lowest energy GB structures (Fig. 8c). The solid black lines represent the fitted mean calculated using Eq. (4a) and the shaded regions in gray correspond to the 95% confidence interval obtained using the diagonal entries in the covariance matrix given by Eq. (4b). The atomistic data points in red (blue) represent the training (testing) points. The predicted values for the segregation energy are cross-validated with the true atomistic simulation data in the testing set, and the results are shown in the insets in Fig. 8b, c. In these insets, GPR model predictions are plotted along the vertical axis and the true atomistic data are plotted on the horizontal axis. The 45$^{\circ }$ line indicates perfect model predictions and is plotted to guide the eye. It can be seen that the data points closely follow the 45$^{\circ }$ line indicating that the GPR model provides a robust fit of GB segregation energy across all testing data points. The calculated RMSE for the model using all GB metastable structures is 0.09, and that for the GPR fit using only lowest energy GB structures is 0.07.

The GPR results in Fig. 8b, c show preferential Mg segregation (i.e., negative GB segregation energy) with increasing $\Delta V/V_o$. This suggests that the use of $\Delta V/V_o$, or equivalently a characteristic distance, as a structural descriptor of the local atomic environment provides a robust fit to the GB segregation data for all metastable structures and GB misorientations. This is attributed to a general feature of the Embedded Atom Method^61^ and Finnis-Sinclair^62^ family of semi-empirical inter-atomic potentials, including the one used in this work^45^, where radially symmetric functions are used to describe the total energy of an atomistic system. It is also interesting to note that the GPR fitted mean in both Fig. 8b, c shows a steeper slope when $\Delta V/V_o < 0$, i.e., regions where the average distance between the Mg atom and its neighbors is smaller than the corresponding ones in the bulk crystal. This can be explained by the asymmetry in the potential energy vs distance functions used in classical inter-atomic potentials, in which functions with steep gradients are used to describe short-range repulsive interactions and ones with long tails describe long-range attractions. As a result, large repulsive energies arise in regions with negative $\Delta V/V_o$ values. The shaded gray regions in Fig. 8b, c represent the 95% confidence, i.e., credible, interval that results from the GPR fit of the GB segregation energy. With our GPR model, predictions of segregation energy $\Delta E$ at each $\Delta V/V_o$ are assumed to be normally distributed, and the scatter in the data at a given $\Delta V/V_o$ will influence both the predicted mean and variance at that point. The total variance of the GPR fit using all GB metastable structures (Fig. 8b) is $\approx 0.10^2$, compared to $\approx 0.06^2$ when using the lowest energy GB structures (Fig. 8c). This suggests that GB metastability serves as a source of variability, leading to larger variance in GPR predictions of segregation energy compared to the case when using only lowest energy structures.

## Conclusions 

The segregation of alloying elements to GBs has been a subject of active research in recent years, for its relevance to many GB-related phenomena, including boundary migration and microstructural evolution, GB diffusion, and mechanical properties. While many experimental and computational studies explored GB solute segregation in a wide range of metallic alloys, the role of GB metastable structures in solute segregation remains poorly understood. Recently, researchers have employed ML techniques to examine GB properties^63–65^. Huber et al.^63^ performed a high-throughput computational study of the segregation of six elemental species to various GB types. The atomistic data were used to arrive at a set of machine learning descriptors to quantify GB segregation distributions. A linear model was developed relating the GB segregation energy to the excess volume and change in coordination number. The study by Wagih et al.^65^ extracted structural features from atomistic simulations of GB segregation in a polycrystalline ensemble of a wide range of alloys by fitting radial basis functions and spherical harmonics to particle densities. The data was then used to construct a linear regression model of the GB segregation energy. Such studies; however, do not systematically examine the role of GB metastable structures in solute segregation, nor do they quantify variability due to metastability effects. In this work, atomistic simulations and GPR modeling were used to address the following questions: To what degree does GB metastability influence boundary segregation? How does solute segregation to metastable GB structures depend on the local atomic environment? If metastability effects are treated as a source of variability in the corresponding segregation energies, how do we quantify such effects? Our atomistic and GPR ML studies exploring various GB misorientation angles (i.e., eleven [001] and eleven [110] STGBs) and site dependency show that GB metastability introduces a plethora of local atomic environments, thereby influencing both the mean and variance of GPR predictions of GB segregation energy. For the Al–Mg system explored in this work, it was found that the GB segregation energy decreases with increasing $\Delta V/V_o$, see Fig. 8, suggesting that Mg segregation to Al GBs is elastically driven, at least in the dilute limit.

Finally, the use of GPR to model GB segregation presents many advantages. First, GPR provides the ability to add prior knowledge about GB segregation and update predictions in a Bayesian learning approach if more data about metastable structures are obtained. Second, with our GPR ML modeling framework, GB metastability is treated as a source of variability, and the boundary segregation energy is expressed as a distribution with a predicted mean [Eq. (4a)] and variance [Eq. (4b)]. In broad terms, our GPR ML approach has many implications beyond the impact of GB metastability on solute segregation. In many microstructure formation and evolution problems, it is typically assumed that GB properties are single-valued quantities that are obtained using ground-state boundary structures. GPR provides a framework, in which GB metastability can be accounted for by expressing boundary properties as distributions with prescribed statistics.

## Supplementary information

Supplementary Information.
