# Supplementary material for: Atomistic and machine learning studies of solute segregation in metastable grain boundaries
Source: Sci Rep. 2022 Apr 23;12:6673. doi: 10.1038/s41598-022-10566-5 (PMC9035190; doi:10.1038/s41598-022-10566-5)
Supplement: Supplementary file 1 — Supplementary Information. [file 41598_2022_10566_MOESM1_ESM.pdf]

Atomistic and Machine Learning Studies of Solute Segregation in Metastable Grain Boundaries

Yasir Mahmood[1], Maher Alghalayini[1], Enrique Martinez Saez[1,2], Christiaan J. J. Paredis[3], Fadi Abdeljawad[1,2]

[1] Department of Mechanical Engineering, Clemson University, Clemson, SC, 29634, USA

[2] Department of Materials Science and Engineering, Clemson University, Clemson, SC, 29634, USA

[3] Department of Automotive Engineering, Clemson University, Greenville, SC, 29634, USA

Table S1. Dimensions of the simulation box for the bicrystal systems employed in the current work. Here, L_y is the dimension normal to the GB plane, and L_x and L_z are the in-plane ones. The dimensions of the simulation box and number of atoms represent the average of all the metastable systems for a given Σ value.

**[001] GBs**					
Σ	$y^{[hkl]}_{upper}/y^{[hkl]}_{lower}$	L_x (Å)	L_y (Å)	L_z (Å)	*Total No. of Atoms*
113	$[15\ 1\ 0]/[15\ \bar{1}\ 10]$	121.63	487.27	20.23	72330
37	$[7\ 5\ 0]/[5\ 7\ 0]$	104.40	418.30	20.23	53273
17	$[4\ 1\ 0]/[4\ \bar{1}\ 0]$	116.75	401.04	20.23	57068
53	$[7\ 2\ 0]/[7\ \bar{2}\ 0]$	117.80	412.53	20.23	59330
29	$[5\ 2\ 0]/[5\ \bar{2}\ 0]$	108.92	392.99	20.23	52213
29	$[7\ 3\ 0]/[7\ \bar{3}\ 0]$	123.23	431.63	20.23	64890
5	$[3\ 1\ 0]/[1\ \bar{3}\ 0]$	115.13	410.14	20.23	57623
53	$[9\ 5\ 0]/[9\ \bar{5}\ 0]$	124.95	416.94	20.23	63525
89	$[8\ 5\ 0]/[8\ \bar{5}\ 0]$	152.65	382.43	20.23	71190
25	$[4\ 3\ 0]/[4\ \bar{3}\ 0]$	121.36	405.20	20.23	59955
257	$[17\ 15\ 0]/[17\ \overline{15}\ 0]$	183.43	550.84	20.23	123370
**[110] GBs**					
Σ	$y^{[hkl]}_{upper}/y^{[hkl]}_{lower}$	L_x (Å)	L_y (Å)	L_z (Å)	*Total No. of Atoms*
73	$[1\ \bar{1}\ 12]/[\bar{1}\ 1\ 12]$	103.69	391.35	28.60	70028
33	$[1\ \bar{1}\ 8]/[\bar{1}\ 1\ 8]$	92.95	329.61	28.60	52800
19	$[1\ \bar{1}\ 6]/[\bar{1}\ 1\ 6]$	88.16	300.29	28.60	45575
9	$[1\ \bar{1}\ 4]/[\bar{1}\ 1\ 4]$	72.81	309.25	28.60	38865
57	$[2\ \bar{2}\ 7]/[\bar{2}\ 2\ 7]$	129.57	306.40	28.60	68430
33	$[2\ \bar{2}\ 5]/[\bar{2}\ 2\ 5]$	98.59	372.80	28.60	63345
43	$[3\ \bar{3}\ 5]/[\bar{3}\ 3\ 5]$	112.54	318.91	28.60	61950
17	$[3\ \bar{3}\ 4]/[\bar{3}\ 3\ 4]$	83.40	331.11	28.60	47625
43	$[5\ \bar{5}\ 6]/[\bar{5}\ 5\ 6]$	132.63	375.80	28.60	86050
27	$[5\ \bar{5}\ 2]/[\bar{5}\ 5\ 2]$	105.10	357.63	28.60	64775
129	$[8\ \bar{8}\ 1]/[\bar{8}\ 8\ 1]$	129.95	367.76	28.60	82500

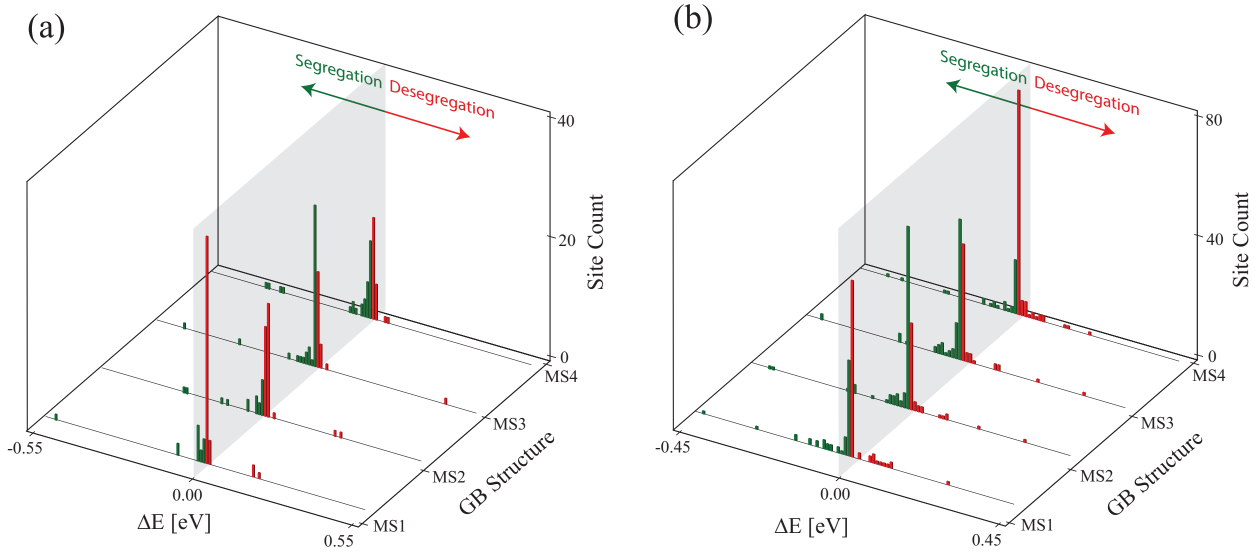

Figure S1. Histograms of the number of Mg segregation sites for the four structures of the (a) Σ5 [001] (310) and (b) Σ19 [110] (116) STGBs. The shaded plane in gray marks the zero segregation energy plane. Regions in green (red) indicate segregation (desegregation).
